# Sensory System for Implementing a Human—Computer Interface Based on Electrooculography

**DOI:** 10.3390/s110100310

**Published:** 2010-12-29

**Authors:** Rafael Barea, Luciano Boquete, Jose Manuel Rodriguez-Ascariz, Sergio Ortega, Elena López

**Affiliations:** Department of Electronics, University of Alcalá, Alcalá de Henares 28871, Madrid, Spain; E-Mails: luciano.boquete@uah.es (L.B.); jmra@depeca.uah.es (J.M.R.-A.); sergioortegarecuero@hotmail.com (S.O.); elena@depeca.uah.es (E.L.)

**Keywords:** electrooculography, eye movement, human–computer interface, wavelet transform, neural network

## Abstract

This paper describes a sensory system for implementing a human–computer interface based on electrooculography. An acquisition system captures electrooculograms and transmits them via the ZigBee protocol. The data acquired are analysed in real time using a microcontroller-based platform running the Linux operating system. The continuous wavelet transform and neural network are used to process and analyse the signals to obtain highly reliable results in real time. To enhance system usability, the graphical interface is projected onto special eyewear, which is also used to position the signal-capturing electrodes.

## 1. Introduction

Much research is under way into means of enabling the disabled to communicate effectively with a computer [1,2], as development of such means has the potential to enhance their quality of life considerably. Depending on users’ capabilities, systems such as speech recognition, brain-computer interfaces [3], and infrared head-operated joysticks [4], *etc.* may be employed for this purpose.

For users with sufficient control of their eye movements, one option is to employ a gaze-direction detection system to codify and interpret user messages. Eye-movement detection interfaces may be based on videooculography (VOG) [5], infrared oculography (IORG) [6] and electrooculography (EOG). Furthermore, this type of interface need not be limited to severely disabled persons and could be extended to any individual with sufficient eye-movement control.

EOG is a widely and successfully implemented technique and has proven reliable and easy to use in human–computer interfaces (HCI). Gips *et al.* present an electrode-based device designed to enable people with special needs to control a computer with their eyes [7]. Barea described an HCI based on electrooculography for assisted mobility applications [8]. The paper studies the problems associated with using EOG to control graphical interfaces and proposes an electrooculographic ocular model to resolve these issues. It also discusses graphical interfaces’ various access options (direct, scanning, gestures, *etc.*). Zheng *et al.* describe an eye movement-controlled HCI designed to enable disabled users with motor paralysis and impaired speech to operate multiple applications (such as communication aids and home automation applications) [9]. Ohya *et al.* present development of an input operation for the amyotrophic lateral sclerosis communication tool utilizing EOG [10]. Bulling *et al.* describe eye-movement analysis for activity recognition using electrooculography [11]. EOG-based systems have also been developed in the robotics field to control mobile robots [12,13] and guide wheelchairs [14,15].

Various techniques may be used to model the ocular motor system using EOG and detect eye movements. These include saccadic eye-movement quantification [16], pattern recognition [17], spectral analysis [18], peak detection deterministic finite automata [19], multiple feature classification [20], the Kalman filter [21], neural networks [22–25] and the support vector machine [26]. Notable efforts have also been made to reduce and eliminate the problems associated with gaze detection in EOG, such as drift, blink, overshoot, ripple and jitter [12,27].

Recent new research has focused on using electrooculograms to create efficient HCIs [18,28,29] and developing novel electrode configurations to produce wearable EOG recording systems, such as wearable headphone-type gaze detectors [21], wearable EOG goggles [17,30,31], or light-weight head caps [32].

The electrooculographic biopotential value varies from 50 to 3,500 μV with a frequency range of about DC-100 Hz. This signal is usually contaminated by other biopotentials, as well as by artefacts produced by other factors such as the positioning of the electrodes, skin-electrode contact, head and facial movements, lighting conditions, blinking, *etc.* To minimize these effects, the system requires high-quality signal acquisition hardware, and suitable analysis algorithms need to be applied to the signal. Signal processing is usually performed on a personal computer. However, a more economical option, and one that also consumes less electricity, is to use a microcontroller-based system.

**Figure 1.**
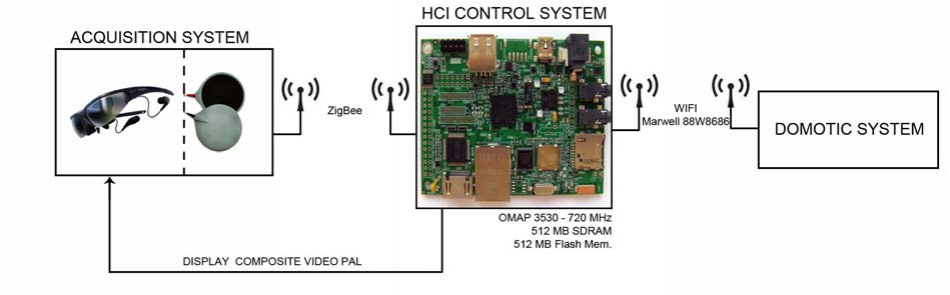
System architecture.

The purpose of this research paper is to develop a system to capture and analyse EOG signals in order to implement an HCI, as shown in Figure 1. The system comprises two electronic modules—the signal Acquisition Module (AM) and the Processing Module (PM). Eyewear incorporating a set of appropriately positioned dry electrodes captures the EOG signals, which the AM acquires, digitizes and transmits using the ZigBee protocol.

The PM receives the signals from the AM and executes the algorithms to detect the direction of the user’s gaze. Simultaneously, it projects the user interface onto the eyewear and, according to the selection made by the user, transmits the commands via WiFi to a home automation system or performs other tasks (*i.e.*, call a nurse, *etc.*).

This paper comprises seven sections. Section 2 describes the signal acquisition and ZigBee-enabled transmission circuit (AM), Section 3 describes the PM, Section 4 describes signal processing, and Sections 5, 6 and 7 present the results, discussion and conclusions of this paper.

## 2. Acquisition System

### 2.1. Wearable EOG Goggles

**Figure 2.**
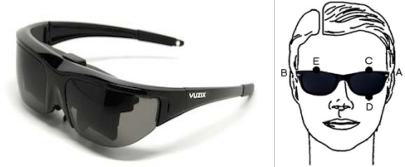
EOG goggles based on Vuzix Wrap 230 eyewear.

The eyewear, which is based on a commercially available model (Vuzix Wrap 230) [33] and features integrated electrodes, performs two functions—it holds the dry electrodes used to capture the EOG signal in position and serves as the medium onto which the user interface is projected. The electrooculogram is captured by five electrodes placed around the eyes. The EOG signals are obtained by placing two electrodes to the right and left of the outer canthi (A–B) to detect horizontal movement and another pair above and below the left eye (C–D) to detect vertical movement. A reference electrode is placed above the right eye (E). The eyewear has a composite video input (PAL format) and displays high-colour, high-contrast images at 320 × 240 resolution, equivalent to a 46-inch screen viewed at a distance of 3 metres. Figure 2 shows the placement of the electrodes in the eyewear.

### 2.2. Acquisition System

The EOG signal is influenced by several factors, including eyeball rotation and movement, eyelid movement, and various artefact sources (electrode placement, head and facial movement, lighting, *etc.*). As the shifting resting potential (mean value) changes, it is necessary to eliminate this value. To do so, an AC high-gain differential amplifier (1,000–5,000) is used, together with a high-pass filter (0.05-Hz cut-off frequency), a relatively long time constant, and a low-pass filter (35-Hz cut-off frequency). The signals are sampled 100 times per second.

**Figure 3.**
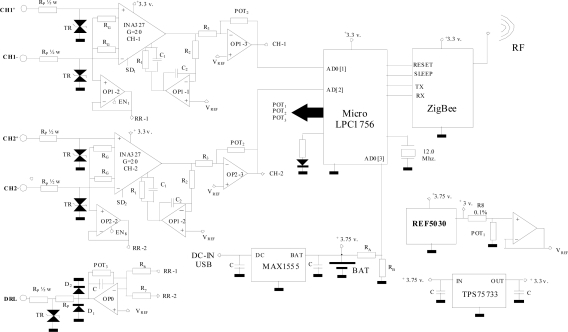
Electrical system diagram.

A two-channel amplifier has been designed and developed to capture bioelectric signals and transmit them using the ZigBee protocol (wireless). This small, portable system’s power supply has been optimized to enable battery-powered operation. One of its main advantages is its versatility, since it enables each channel to be configured dynamically and individually (active channel adjustment, channel offset, gains, sampling frequency or driven-right-leg circuit gain) via commands sent over the ZigBee protocol. Figure 3 shows the electrical system diagram for data capture, amplification, digitization and transmission.

The analogue signal acquisition hardware comprises two differential inputs (CH1, CH2), which are digitized by the microcontroller's internal ADC (LPC1756, 12-bit resolution, 100–300 Hz sampling frequency adjustable in 10-Hz steps) before being transmitted via the ZigBee protocol.

The system communicates via the ZigBee protocol, acting directly on the link level (802.15.4). Due to its low energy consumption and widespread implementation in low-cost commercial systems, this protocol is considered the best option. The XBee module is connected to the microcontroller by D_OUT_ and D_IN_ lines. The XBee module and the microcontroller communicate via an 115,200 bps serial connection and the XBee module is controlled by API frames. The XBee device’s command set allows users to configure the network and serial interface parameters via the microcontroller. Although this paper considers ZigBee the best option because of its low energy consumption, the electrical system diagram can be easily modified to implement Bluetooth, which is a much more widely used communications standard, although it requires considerably more power.

The device is powered by rechargeable lithium-ion batteries (3.75 V DC/6.8 Ah). Consumption has been reduced by using integrated circuits with a shutdown (SD) feature, which means they can be deactivated when not needed by the active application. The batteries are recharged from a computer USB port using a MAX1555 integrated circuit.

The integrated circuits have a 3.3-V power supply provided by regulator TPS75733, which draws power directly from the battery. The circuit uses a very stable 3.0-V reference voltage (REF5030). A digital potentiometer (POT_1_:MCP4261) is then used to transform this into a variable reference voltage (V_REF_). This potentiometer is adjusted via the microcontroller using the serial peripheral interface (SPI) protocol and is used to configure each channel.

The amplification margin of the signals recorded by the two channels is established independently (0–5,000 adjustable gain). The lower cut-off frequency is set at 0.05 Hz and the upper cut-off frequency is set at 35 Hz.

One of the aspects bearing most heavily on final system quality is front-end amplification of the bioelectrical signal. In many cases, the bioelectrical signals’ amplitudes are below 50 μV and are usually contaminated by various noise sources, such as the network alternate component (50 or 60 Hz and its harmonics), electrode contact noise (baseline drift), other physiological patient systems (*i.e*., muscular noise), interference from electronic devices, *etc.* To minimize these effects, various techniques may be used to optimize analogue signal capture. The proposed architecture employs two-stage signal amplification. The first stage comprises a differential amplifier (G_1_ = 20 = R_1_/R_G_) based on an instrumentation amplifier (INA327) with a shutdown feature (SD_i_), which enables unused channels to be deactivated. This first stage’s general gain expression is shown below:

(1) G1(s)=R1RG1+s.R1.C1.s.C2.R21+s.C2.R2

This amplifier has a mean frequency gain of R_1_/R_G_, a lower cut-off frequency of 0.05 Hz (2π R_2_.C_2_)^−1^ and an upper cut-off frequency of (2π R_1_.C_1_)^−1^. The upper cut-off frequency of the input stage is set by the R_1_.C_1_ product. As R_1_ should be kept constant to avoid modifying the amplifier gain, C_1_ has been modified to produce an upper cut-off frequency of 35 Hz.

The second amplification stage uses the OPA2334 operational amplifier, which has been chosen for its low offset voltage. As it is an adjusted gain inverting amplifier (G_2_ = −POT_2_/R_3_), the gain can be set between 0 (POT_2_ resistance = 0 Ω) and −250 (256 steps), while signal amplification allows total gain per channel of 0–5,000.

**Figure 4.**
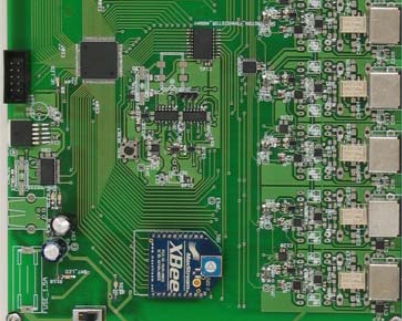
Image of the AM.

The Driven-Right-Leg (DRL) circuit allows common mode signal reduction, applying a circuit feedback voltage to the patient [34]. Each channel's common mode signal is captured by a voltage follower (OPi-2 = OPA2334) and another similar amplifier adds them together. The feedback circuit gain can be adjusted by commands from the host using POT_3_ (MCP4261) on each acquisition channel. This improves signal capture, as the patient’s potential value depends on many factors (electrode location, proximity to the feedback network, stretcher type, *etc.*). Figure 4 shows the implemented AM.

## 3. Processing Module

The PM's function is to receive the EOG signals via the ZigBee protocol, apply the appropriate algorithms to detect the user’s eye movements, display the user interface on the eyewear, decodify the user's message, and send the appropriate command via WiFi to the home automation system that will execute the user’s instructions (switch on TV, *etc.*). In addition, during the training and calibration phases, the PM captures the user’s eye movements and with them trains a radial-basis-function (RBF) neural network using the Extreme Learning Machine (ELM) algorithm. It then sends the commands to the AM to adjust the system’s operating parameters (amplifier gain, offset, *etc.*).

The PM is based on a high-performance SoC (System on Chip), the OMAP3530, which includes a Cortex-A8 core as well as a C64x + DSP running at 720 MHz. It has 512 MB of RAM and 512 MB of flash memory. It provides a direct composite video output (compatible with the PAL and NTSC formats) connected to the Vuzix Wrap 230 eyewear.

The operating system used by the processing card is an OpenEmbedded-based Linux distribution optimized for the ARMv7 architecture with the ARM/Linux kernel (version 2.6.32) and the U-Boot 1.3.4 bootloader. The OpenEmbedded-based file system includes the XFCE-lite graphic environment. Using a Linux environment provides access to a multitude of graphic and console applications and utilities. The system has the capacity to compile its own programs as it includes the GCC compiler and auxiliary native tools (Binutils).

The PM communicates with the AM via a ZigBee link. To achieve this, two commercial ZigBee modules (XBee) are connected to the respective UARTs on the processing (OMAP3530) and acquisition (LPC1756) cards' microcontrollers. Communications via the ZigBee protocol are performed with a power level of 0 dBm.

Data acquisition via the ZigBee protocol is performed constantly and in real time. To achieve this, a high-priority process scans the buffer of the UART connected to the ZigBee module and sends these data to the two processes responsible for executing the signal-processing algorithms (wavelet transform and neural network).

Once the user’s eye movements in relation to the user interface projected onto the eyewear have been decodified and interpreted, the orders are sent via WiFi (because of its universality) to a home automation system or similar application. The WiFi interface is implemented using a Marvell 88W8686 (IEEE 802.11 b/g) chipset connected to the OMAP3530. The WiFi stack is part of the ARM/Linux 2.6 kernel (Linux wireless subsystem, IEEE-802.11) and includes the necessary wireless tools (iwconfig, iwlist, *etc*).

## 4. Signal Processing

**Figure 5.**
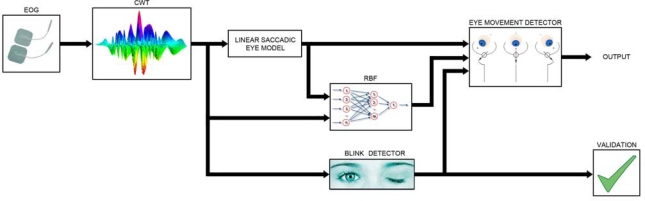
Saccadic eye-movement detection process.

Figure 5 shows the processing performed on the digitized EOG signal. Processing is structured into two phases. The first phase is optional and consists of adjusting the signal-capture system, applying the linear saccadic eye model and training the neural network according to the user’s signals. This option can be activated when a new user utilizes the system or when the user’s responses change due to tiredness or loss of concentration. Parameter adjustment should be performed by someone other than the user. The acquisition module allows for adjustment of channel gain, DRL gain and the V_REF_ parameter. The ocular model calculates the relationship between EOG variation and the eye movement performed, as well as calculating the minimum detection threshold.

Once appropriate signal-capture conditions are established, the system instructs the user to look at a series of pre-determined positions on the user interface. The EOG signal is filtered by the wavelet transform and a linear saccadic model is used to detect and quantify saccadic movements. These signals are then used as samples to train the neural network. The neural network’s purpose is to enhance detection of saccadic movements by using pattern recognition techniques to differentiate between variations in the EOG attributable to saccadic movements and those attributable to fixation problems or other artefacts. As users become tired and their concentration deteriorates (particularly after long periods of operation), these artefacts in the EOG signal become increasingly pronounced.

The continuous wavelet transform (CWT) is useful for detecting, characterizing and classifying signals with singular spectral characteristics, transitory content and other properties related to a lack of stationarity [35]. In the case addressed here, the best results were obtained by using the db1 mother wavelet from the Daubechies family due to its strong correlation with the changes the system aims to detect in the original EOG signal. The CWT makes use of modulated windows of variable size adjusted to the oscillation frequency (*i.e*., the window's domain contains the same number of oscillations). For this reason, the method employs a single modulated window, from which the wavelet family is obtained by dilation or compression:

(2) $$\Psi_{b,a}\;(t)\to \frac{1}{\sqrt{\left|a\right|}}\Psi \left(\frac{t-b}{a}\right)$$

where *a* ≠ 0 and b are the scale and latency parameters, respectively. The energy of the functions is preserved by a normalized factor $1/\sqrt{\left|a\right|}$.

The optimal scale that produced greatest correlation in the studies carried out was a = 60. The effect of this wavelet is similar to that of deriving the signal (high-pass filtering), although the results are magnified and it is easier to identify saccadic eye movements as the threshold is not as critical.

The linear saccadic model considers that the behaviour of the EOG is linear. This is equivalent to stating that the eye movement is a constant of the variation of the EOG (eye movement = k*EOG_variation) [8]. A saccadic movement is considered to occur when the EOG derivative exceeds the minimum threshold. The direction and size of a saccade is given by its sign and amplitude. The neural network implemented is a radial-basis-function (RBF) network trained using the ELM algorithm [36], which is characterized by its short computational time. The network’s input data comprise the contents of a 50-sample time window (25 preceding samples and 25 subsequent samples) from the EOG signal corresponding to a detected eye movement and are processed using the wavelet transform. The internal structure has 20 neurons in the hidden layer. The network’s output determines whether a valid saccadic movement has occurred. Network training is performed on a set of 50-sample segments taken from the EOG at different instants. These correspond to resting (gaze directed at the centre), saccadic eye movements, and fixation periods. Output is “1” when a saccadic movement exists and “0” in all other cases. The output of the neural network is a linear combination of the basis functions:

(3) $$f(x)=\sum_{i=1}^{N_{H}}\beta_{i}.\text{exp}(-\frac{{\left\|w_{i}.x-\mu_{i}\right\|}^{2}}{\sigma_{i}^{2}})=\sum_{i=1}^{N_{H}}\beta_{i}.g_{i}(x)$$

where β_i_ denotes the output weight matrix, w_i_ are the input weights and σ_i_ is the width of the basis function.

The ELM algorithm is a learning algorithm for single hidden-layer feed-forward networks. The input weights (w_i_), centres (μ_i_) and width of the basis function are randomly chosen and output weights (β_i_) are analytically determined based on the Moore–Penrose generalized inverse of the hidden-layer output matrix. The algorithm is implemented easily and tends to produce a small training error. It also produces the smallest weights norm, performs well and is extremely fast [37].

**Figure 6.**
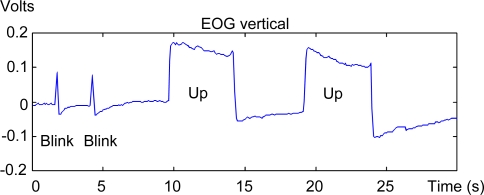
Effect of blinking on the vertical EOG.

A block has also been designed to work in a similar way to a mouse click to enable users to validate the desired commands. This block detects two or three consecutive blinks within a time interval configured according to the user’s capabilities. Blink detection is based on pattern recognition techniques (a blink template is created from user blink segments). Blinks are detected by comparing the template against the EOG’s vertical component. A blink is considered to exist when there is a high level of similarity (above a pre-determined threshold) between the template and the EOG’s vertical component. Figure 6 shows an example of the difference on the vertical EOG between blinking and an upward saccadic movement. As may be seen in the EOG recorded in each case, the duration of the blink is shorter than that of the saccadic movement.

Finally, based on the neural network, linear saccadic model and blink detector outputs, the eye-movement detector block determines the validity of the saccadic movement detected. When the linear saccadic model detects a saccadic movement, a 50-sample window from the EOG signal (centred on the instant the saccadic movement is detected) is input into the neural network. The network output determines the movement’s validity. Meanwhile, to eliminate the blink effect on the EOG signal, when a blink is detected, the saccadic movement detected at the same instant is discarded. Furthermore, as the neural network’s training segment is longer than a blink segment, the effect can be filtered immediately by the neural network to remove false saccadic movements.

**Figure 7.**
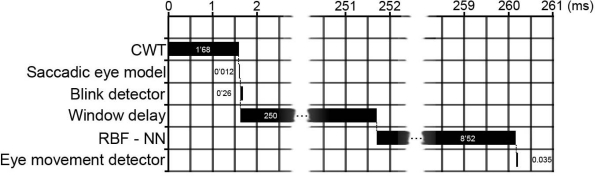
Timeline.

As regards the system's computational time, a 260.49 ms delay exists between performance of a saccadic movement and its validation. This delay may be considered appropriate for typical graphical interface control applications. Figure 7 shows a timeline displaying the various processing stage times. The EOG signals are sampled 100 times per second. 1.68 ms are needed to process the CWT, while the linear saccadic model takes 0.012 ms to detect the movement and quantify it. Blink detection takes 0.26 ms. A 250 ms delay is needed after a saccadic movement is detected (corresponding to 25 subsequent samples from the EOG signal) before the signal can be propagated over the neural network (50 samples). Signal propagation over the RBF takes 8.52 ms. Finally, the eye-movement detector block requires 0.035 ms to validate the movement performed.

**Figure 8.**
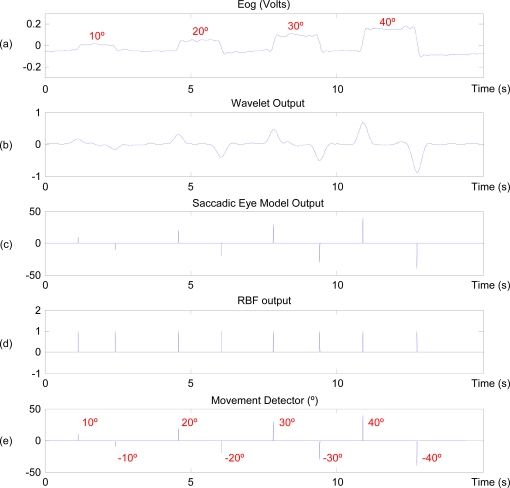
Eye-movement detection process sequence.

Although this may appear to be a long delay, it only occurs when a saccadic movement is detected. In all other cases, the signal is not propagated over the RBF and the system's computational time stands at 1.975 ms. Given that the system acquires a sample every 10 ms, to all practical intents and purposes it processes the EOG signal in real time. The second phase comprises a cyclical process in which the EOG is captured and the signals are processed to determine which command the user wishes to activate. Figure 8 shows an example of processing of a typical horizontal-channel signal—the user’s gaze progressively shifts 10, 20, 30 and 40 degrees horizontally [Figure 8(a)]. First, the EOG signal is filtered using a CWT [Figure 8(b)], then the linear saccadic model detects saccades and determines their angle [Figure 8(c)]. When a movement is detected, the EOG signal is input into the neural network, which determines if it is an eye movement [Figure 8(d)]. Finally, based on the neural network and blink detector outputs, the eye-movement detector determines the validity of the saccadic movement. It then quantifies the movement according to the value obtained in the linear saccadic model [Figure 8(e)].

The system developed is able to detect eye movements to within an error of 2 degrees, making it possible to select or codify a large number of commands within a particular graphical interface. Furthermore, the validation block makes it possible to validate the command selected or eye movement performed.

## 5. Results

This paper implements a prototype wearable HCI system based on electrooculography. The eyewear is used to position the electrodes and display the user interface, thereby facilitating system usability. Signal capture is performed by a low-power-consumption electronic circuit. The prototype's intelligent core is based on a high-performance microcontroller that analyses the signals and transmits the user's commands to a home automation system via a WiFi connection.

Eye movement-based techniques employed to control HCIs include Direct Access, Scanning and Eye Commands (gestures). Direct Access is the most widely used form (in which the user, when shown a graphical interface, selects the desired command by positioning a cursor over it and then carrying out a given validation action, usually a mouse click). If the graphical interface is vision-controlled, the cursor is directed by eye movements and validation is performed either on a time basis or by an ocular action such as blinking. The drawback of this interface is the ‘Midas Touch’ problem, as the human eye is always active. Therefore, it is necessary to ensure that validation cannot be performed involuntarily. To avoid this problem, eye-movement codification is generally used. The aim of this technique is to develop control strategies based on certain eye movements (ocular actions or gestures) and their interpretation as commands. Usually, eye-movement recognition is based on detecting consecutive saccades, which are then mapped to eye movements in basic directions—left, right, up and down [18,19,29,38].

As quality in graphical interface control is partly measured in terms of ease-of-use and system simplicity, the Direct Access technique was selected as it is the most natural and fastest and, therefore, the most comfortable to use. Furthermore, it also allows the system to include a large number of commands without the need for users to memorize complex ocular actions.

To operate the system, the user looks at the centre of the screen and then looks at the desired command (saccadic movement). This selects the command, which is then validated by two consecutive blinks within a pre-determined time limit, which starts when the eye movement commences and is configured according to the user’s capabilities.

**Figure 9.**
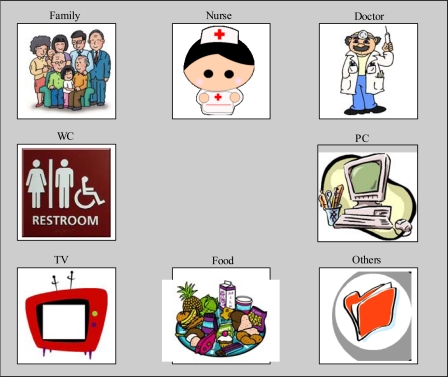
Eight-command interface.

As the system detects the eye-movement angle to within an accuracy of 2 degrees, it is technically possible to design an interface containing a large number of commands. However, experience shows that a simpler interface with 4–8 commands is preferable, as the capabilities of users likely to operate these interfaces need to be taken into account. This means detecting simple up, down, left and right movements and their corresponding diagonals. Figure 9 shows one of the interfaces implemented.

The system was tested by five volunteers (three men and two women) aged between 22 and 40 using an 8-command user interface. Thirteen 5-minute tests were performed per volunteer (1 hour in total). The tests required volunteers to select each of the interface’s commands cyclically (13 × 5 × 8 = 520 selections in total, 104 per volunteer). Once a saccadic movement was detected, the user had 2 seconds to perform validation (double blink).

Training for volunteers to familiarize themselves with the system took approximately 5 minutes and during this time a member of the research group calibrated the system (gain, offset, *etc.*) for each volunteer. The system then trained the neural network using the real data captured from each volunteer.

**Figure 10.**
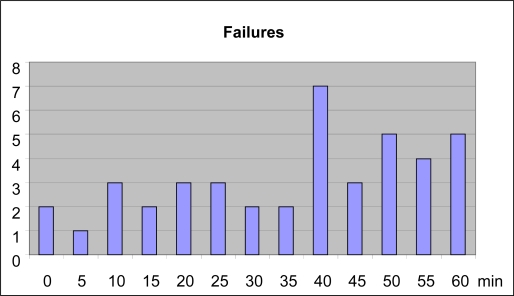
HCI errors in relation to time.

As table 1 shows, the volunteers achieved an overall success rate of 92%. The errors produced were due to problems in either saccadic movement detection (66% of errors) or command validation (34% of errors). Figure 10 shows the distribution of these failures by test.

The following may be concluded from the results obtained:

- The number of failures is low initially because user concentration is high. Providing prior training improved these results, as the user was already accustomed to operating the HCI and, furthermore, the neural network was trained on each user's own signals.
- The number of failures increases with time, a trend principally attributable to falling user concentration and increasing tiredness. However, the number of failures is much lower than when using other electrooculographic models [8].

These results, which naturally may vary according to users’ physical and mental capabilities, preliminarily demonstrate that the system implemented operates as intended.

## 6. Discussion

Much of recent research into EOG-based HCI systems focuses on (a) developing wearable systems, and (b) enhancing system reliability by implementing new processing algorithms. This paper presents advances in both regards. On the one hand, it implements a modular hardware system featuring wearable goggles and a processing unit based on a high-performance microcontroller and, on the other, the EOG signal-processing technique employed provides satisfactory HCI control.

This paper uses a continuous wavelet transform to filter the EOG signal and employs a neural network to provide robust saccadic-movement detection and validation. The system has been validated by 5 healthy users operating a Direct Access eight-command HCI.

Initial EOG signal processing using the continuous wavelet transform enhances the non-stationary and time-varying EOG signal [39] and therefore, in our case, makes identification of smaller saccadic movements possible. This is vitally important when working with Direct Access interfaces that require highly accurate eye-movement detection. In this paper, the best results were obtained using the db1 mother wavelet from the Daubechies family at scale 60. Other papers have employed the continuous 1-D wavelet coefficients from the signal at scale 20 using the Haar wavelet [30]. This paper’s authors obtained better results with the Daubechies mother wavelet than with the Haar mother wavelet or with conventional or adaptive filtering techniques (Wiener filter, *etc.*) [8].

Neural networks have long been used successfully to process EOG signals [22,23]. Various training algorithms and architectures have been researched and have produced generally satisfactory results [24,40,41]. Both the neural network architecture used in this case (RBF), and its training algorithm (ELM), were optimized for real-time use on a microcontroller-based system.

As commented in the Results section, eye movement-based techniques employed to control HCIs include Direct Access, Scanning and Eye Commands (gestures). Direct Access is the most widely implemented technique because it is the most natural and the fastest and, therefore, the most comfortable to use. Furthermore, it also allows the system to include a large number of commands without the need for users to memorize complex ocular actions. Drawbacks associated with this interface include the ‘Midas Touch’, eye jitter, multiple fixations on a single object, *etc.* To avoid these problems, one widely used option is to develop applications based on eye-movement codification or gestures [16,42]. The system presented in this paper accurately detects all eye movements, which means that the resulting gesture-based HCI (using codified up, down, right and left eye movements) is extremely robust.

As yet, a testbed widely accepted by researchers to measure and compare the results of EOG-based HCI systems does not exist. User numbers and characteristics, user interfaces and experiment length, among other aspects, all vary from paper to paper. In this paper, the tests designed to generate messages valid for a home-automation system performed by five healthy volunteers produced an overall 92% success rate. The authors consider this sufficient to ensure satisfactory user communication. These results are similar to those achieved in other recent papers describing development of applications based on eye-movement codification. Nevertheless, most of these papers show the results obtained when detecting up, down, left and right eye movements, which are significantly easier to detect than other types of eye movement. For example, in a work by Deng *et al.*, 90% detection accuracy is obtained for these movements and the system is used to control various applications/games [38]. In Gandhi *et al.* [19], detection and device-control accuracy is 95.33%. The nearest neighbourhood algorithm is used by Usakli *et al.* to classify the signals, and classification accuracy stands at 95% [28]. In a work by Bulling *et al.*, eye movements are studied to detect gestures used to control a graphical interface. Accuracy (around 90%) is calculated as the ratio of eye movements resulting in a correct gesture to the total number of eye movements performed [43].

However, few papers on EOG quantify movement detection accuracy and those that do quantify it do not employ the same parameters, thereby preventing exhaustive comparison between them. In this paper, the combination of the wavelet transform, the ocular model and the neural network produce a measurement error of less than 2 degrees. This error is in the same order of magnitude as that of other EOG-based HCI systems [44,45]. Other authors, such as Manabe *et al.*, report that the average estimation error is 4.4 degrees on the horizontal plane and 8.3 degrees on the vertical plane [21].

One of the contributions made by this paper is that the neural network eliminates or minimizes the fixation problems that appear when the user becomes tired and that become increasingly significant when the HCI is used for long periods. Comparison between the number of false saccadic movements detected in 60-minute EOG recordings by the linear saccadic model based on derivatives implemented in Barea *et al.* [16] and the architecture proposed in this paper demonstrates that saccadic-movement detection errors due to fixation problems and artefacts derived from blinking have been practically eliminated. It is also noteworthy that although the error obtained in 20, 30 and 40-degree movements is in the order of 2 degrees (similar to that obtained in Barea [8]), a substantial improvement has been produced in detection of small saccadic movements (the error produced in detection of 10-degree saccadic movements has been reduced by 50%). This is principally due to improvement of the S/N ratio by the wavelet in comparison with conventional filtering techniques.

As regards the system’s computational time, this stands at 260.49 ms when a saccadic movement is detected. Command validation time should also be added to this delay. In the system implemented in this paper, the principal bottleneck lies in the neural network, which requires a 500-ms EOG signal window. However, the improvement in result quality and reliability justifies neural network use. Furthermore, in most graphical interface control applications, this delay is not critical and does not affect usage of the system proposed. It should also be underlined that computational time when a saccadic movement is not detected stands at 1.975 ms, which means that to all practical intents and purposes the system works in real time.

The authors propose the following areas for future research:

- System validation by a greater number of users (principally disabled users).
- Study of system performance in mobile settings. Although the results presented in this paper were obtained under static conditions, previous papers have examined conditions in which users were mobile [14]. This paper has developed algorithms to eliminate artefacts generated principally by errors deriving from electrode contact with the user's skin (skin–electrode interface) and facial movements or gestures. However, use of a different electrode type (dry electrodes) and a new method of attaching the electrodes to the user’s face, as well as use of these systems in mobile settings, require in-depth study of the new problems/artefacts that may arise.
- Improvements to system features. On-line self-calibration of the ocular model parameters every time a new saccadic movement is detected. This would enable users to work with the model for long periods without the need for third-party intervention to calibrate the system if adjustment errors were detected.
- On-line neural network training. One of the advantages of using the ELM algorithm is its speed. In the tests performed in this paper, 14.5 ms were needed to train one hundred 50-sample EOG segments. The short training time required makes it possible to perform on-line training every time a new saccadic movement is detected.

## 7. Conclusions

This paper presents a system to capture and analyse EOG signals in order to implement an HCI interface. Specific hardware has been developed to capture users’ biopotentials and a Linux platform has been used to implement the algorithms and graphical user interface. The eyewear employed performs the dual function of capturing the EOG signal comfortably and implementing the user interface.

The results (92% reliability) demonstrate that the system proposed works well and produces an error rate that permits its use as part of an HCI. As the system is portable, it may be easily implemented in home automation, robotic systems or other similar applications. Furthermore, the hardware platform’s processing power provides scope to implement more complex signal-analysis algorithms.

## Figures and Tables

**Table 1.** Experiment results.

	**Successes**	**Failures**
User 1–man	89	15
User 2–woman	98	6
User 3–man	94	10
User 4–woman	100	4
User 5–man	97	7
